# Propagation of Interpreter Errors by Ambient AI Scribes: Study Using Simulated Clinical Encounters

**DOI:** 10.2196/88734

**Published:** 2026-07-28

**Authors:** Alexandra Rabotin, Efren Aguilar, Saitiel Sandoval Gonzalez, Alonso Iniguez, Christina Jung, Eric Lee, Douglas S Bell, Jeffrey Arroyo

**Affiliations:** 1 Family Medicine Residency Program, Mission Community Hospital, 14860 Roscoe Blvd, Panorama City, CA, 91402, United States, 1 818-787-2222; 2 Department of Pathology, Mass General Brigham, Boston, MA, United States; 3 David Geffen School of Medicine at UCLA, University of California, Los Angeles, Los Angeles, CA, United States; 4 Department of Clinical Informatics, AltaMed Health Services, Los Angeles, CA, United States; 5 Division of General Pediatrics, Children's Hospital of Los Angeles, Los Angeles, CA, United States; 6 Keck School of Medicine, University of Southern California, Los Angeles, CA, United States; 7 Department of Medicine, David Geffen School of Medicine at UCLA, University of California, Los Angeles, Los Angeles, CA, United States

**Keywords:** ambient clinical documentation, digital scribes, ambient scribes, ambient AI scribes, medical interpreting, artificial intelligence, speech recognition software, patient safety, communication, documentation

## Abstract

In simulated English and Spanish clinical encounters, ambient AI scribes propagated interpreter errors into clinical notes, with patterns varying by speaker role and error type. These findings highlight the need for further evaluation of AI-scribe performance in multilingual and interpreter-mediated clinical care.

## Introduction

Ambient AI scribes (systems that passively capture clinical conversations and generate structured documentation) are being rapidly deployed despite concerns regarding note quality and accuracy, even in monolingual encounters [1,2]. Given that over 26 million people in the United States have a non-English language preference and that interpreter errors are common [3-7], it is important to evaluate AI scribe performance in interpreter-mediated settings.

To our knowledge, no prior studies have examined how AI scribes handle documentation when interpreter errors are introduced. This study aimed to assess whether AI scribes incorporate interpreter errors into clinical notes.

## Methods

### Overview

We developed 5 scripted Spanish and English clinical scenarios containing a total of 20 deliberate interpreter errors (omissions, substitutions, or additions) in both patient and clinician contexts, modeled on those described in prior studies [4-7].

Three native Spanish-speaking medical students simulated the encounters as patient, clinician, and interpreter (maintaining consistent roles). Simulations were audio-recorded and processed through two ambient AI scribes: vendor A (with explicit Spanish support) and vendor B (which used GPT-4o). Vendors were anonymized to focus the evaluation on identifying systemic vulnerabilities across these platforms rather than benchmarking specific products.

The primary outcome was error propagation, defined as the incorporation of the interpreter’s errors into the AI-generated notes. Two investigators independently reviewed all notes. Error propagation rates were summarized by error type, error context, and vendor. Given the small number of scripted errors, results were summarized descriptively. Additional methods are described in Multimedia Appendix 1.

### Ethical Considerations

The Kansas Health Science University Institutional Review Board, serving as the central review board for Mission Community Hospital, determined that this study does not constitute human subjects research (#KHSC_IRB-2025-12).

## Results

**Figure 1.**
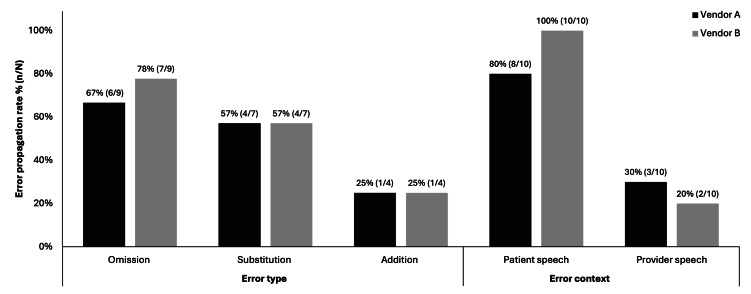
Error propagation by 2 ambient AI scribe vendors, stratified by error type and error context. “Patient speech” refers to English interpretation of Spanish patient statements and also includes the caregiver in pediatric scenarios; “clinician speech” refers to Spanish interpretation of English clinician statements.

Both AI scribes propagated interpreter errors into the clinical note, though rates varied by error type and by context (Table 1, Figure 1). Vendor A propagated 55% (11/20) of interpreter errors, and vendor B propagated 60% (12/20). Omission errors were propagated in 67% (6/9) and 78% (7/9) of cases for vendors A and B, respectively; substitution errors in 57% (4/7) for both vendors; and addition errors in 25% (1/4) for both vendors. Propagation was higher for errors originating in patient speech than for errors originating in clinician speech. For patient-speech errors, vendor A propagated 80% (8/10), and vendor B propagated 100% (10/10). For clinician-speech errors, vendor A propagated 30% (3/10), and vendor B propagated 20% (2/10). Interrater agreement for coding propagated errors was 97.5% (39/40), with the discrepancy resolved through consensus.

**Table 1.** Examples of interpreter errors and their documentation and propagation by 2 ambient AI scribes.

Error type and original statement		Interpreter error	Vendor A documentation	Vendor B documentation	Propagation (vendor A/vendor B)
Omission					
	Patient: “Well… yes, I think *I do not hear that well*. And now that I think about it, yes, sometimes I have a ringing in my right ear; it sounds like an annoying noise.”	“Now that I think about it, I sometimes have a ringing in my right ear; sounds like an annoying noise.”	“They occasionally experience ringing in the right ear, described as an annoying noise, with *no other hearing problems*.”	“The patient also sometimes experiences a ringing in the right ear, which is described as an annoying noise.”	Yes^a^/yes
	Patient: “No fever, but he coughs at night and *wheezes* when he breathes.”	“No fever, but he coughs at night.”	“The patient presents with a persistent cough and nighttime wheezing.”	“The patient presented with nighttime cough…”	No/yes
	Patient: “It started on his arm. He has a *yellow crust* that he keeps scratching…”	“It started on the arm and is very itchy…”	“He has severe itching and yellow crusting on the rash…”	“The patient presented with a persistent and very itchy rash on the arm…”	No/yes
	Clinician: “...I will send you a stronger steroid cream. *Don’t use it more than 10 days*.”	“...I’m going to send a stronger steroid cream.”	“Prescribe stronger topical corticosteroid.”	“Prescribed a stronger steroid cream to be used for no more than 10 days.”	Yes/no
	Clinician: “Have you noticed any *black stools* or bright-red blood in the stool?”	“Have you seen bright red blood in the stool?” (Patient answered “No”)	“He denies black stools or bright red blood in the stool.”	“The patient reported not having black stools or bright red blood in the stool.”	Yes^a^/yes^a^
Substitution					
	Clinician: “...you can take extra strength Tylenol, 2 tablets every *6* hours...”	“...you can take extra-strong Tylenol, 2 tablets every *4* hours…”	“Advise acetaminophen, two tablets every six hours…”	“The patient was advised to take extra strength Tylenol, two tablets every six hours...”	No/no
	Patient: “Sometimes I start to feel *hot* when I feel that way.”	“Sometimes there’s *sweat* when I feel that way.”	“…and sweating occur occasionally…”	“and occasionally caused sweating.”	Yes/yes
	Patient: “He complains that his throat itches.”	“He says his throat is *burning*”	“He also has a burning sensation in the throat.”	“The patient reported a burning sensation in the throat.”	Yes/yes
Addition					
	Clinician: “...just humidifier and honey.”	“just a humidifier, honey, and a *little lemon*”	“Advise using a humidifier and honey….”	“Advised use of a humidifier and honey...”	No/no
	Patient: “Three months ago.”	“*More than* three months ago.”	“…persisted for over three months…”	“…for more than three months…”	Yes/yes

a Primary outcome coding rule and sensitivity analysis. Evaluated from a clinician’s perspective, cases where an interpreter’s omission was associated with the AI scribe documenting an unsupported clinical denial (eg, documenting a denial of a symptom the patient was never actually asked about by the interpreter) were coded as error propagation (rather than hallucination, for example). This reflects how the interpreter error was associated with changing the final clinical end point. If these borderline negative-finding cases were interpreted as nonpropagation, the overall error propagation rates would change from 55% (11/20) to 45% (9/20) for vendor A, and 60% (12/20) to 55% (11/20) for vendor B. The omission error propagation rate would change from 67% (6/9) to 44% (4/9) for vendor A, and from 78% (7/9) to 67% (6/9) for vendor B. Patient speech errors would change from 80% (8/10) to 70% (7/10) for vendor A, and remain unchanged at 100% (10/10) for vendor B. Clinician speech errors would change from 30% (3/10) to 20% (2/10) for vendor A, and from 20% (2/10) to 10% (1/10) for vendor B.

## Discussion

This study provides early evidence that ambient AI scribes may propagate errors into clinical notes. Across 2 vendors, over 50% of scripted interpreter errors appeared in the resulting notes, with a higher propagation rate for errors arising from patient speech than from clinician speech.

Previous work in various clinical settings has demonstrated that interpreter-mediated communication often contains errors, some of which may negatively impact clinical care [4-7]. We are unaware of any previous studies that assessed AI-scribed documentation of interpreter errors. Prior research has highlighted broader concerns about the rapid deployment of AI scribes without evaluation of reported or unknown risks, such as omission errors, speaker misattribution, and disparities in speech transcription between racial and ethnic groups [1,2,8-10]. These risks may be heightened in encounters with medical interpreters, involving multiple speakers and languages.

As shown in Figure 1, even though errors originating in English (clinician speech) were less commonly propagated than those in Spanish (patient speech), we can see instances of propagation and nonpropagation in both languages (across both vendors in aggregate). These findings suggest context-dependent behavior. Several mechanisms may explain this variability. Many foundation models and commercial speech systems are trained predominantly on English data, so differential handling of English and Spanish content remains a plausible contributor. However, this explanation alone does not account for all observed cases. For example, in instances such as the “wheezing” and “yellow crust” scenarios, vendor A successfully recovered the original Spanish-source content and bypassed the erroneous English interpretation. The models may also implicitly weigh some speakers more heavily than others (ie, clinician speech over interpreted speech, and interpreted speech over patient speech). Other factors, including error type, clinical context, audio quality, and vendor-specific processing pipelines, may also influence whether interpreter errors are propagated. These hypotheses require further testing.

Some scenarios challenged our definition of “propagation.” In one case, a medication instruction frequency was altered, yet the AI documented the original schedule (every 6 hours instead of every 4 hours; Table 1). In another case, the omission of “black stools” from the interpreted question led the AI scribe to document a full negative response, suggesting that the model may have relied on source English clinician speech rather than the interpreted exchange. The case of omitting “not hearing well,” which led to documentation of “no other hearing problems,” can be seen as an unsupported negative finding or a hallucination. These examples highlight the need for clearer taxonomies of AI-scribe errors in multilingual encounters.

This study is limited by its small, scripted sample size and the use of recorded audio playback, which limits its generalizability. Additionally, because we evaluated final clinical summaries rather than intermediate raw transcripts, we could not systematically distinguish between automatic speech recognition failures in transcribing Spanish and natural language processing failures in which the model may have discarded Spanish content in favor of an English interpretation. Finally, because generative AI models undergo frequent, unannounced updates, the reproducibility of these findings is inherently limited by the specific model versions active at the time of testing (August 2025). Additional limitations and future-study considerations are described in Multimedia Appendix 2.

Our study suggests that AI scribes may propagate interpreter errors in clinical documentation. Larger studies using more diverse scenarios, language pairs, vendors, and real-world interpreter-mediated encounters are needed to better characterize these risks and guide safe deployment.

## Supplementary material

10.2196/88734 Multimedia Appendix 1 Overview of simulated primary care scenario design, interpreter error classification, acoustic testing environment, and error propagation mechanics.

10.2196/88734 Multimedia Appendix 2 Supplemental information on limitations and suggested considerations for future studies.
