# Supplementary material for: Propagation of Interpreter Errors by Ambient AI Scribes: Study Using Simulated Clinical Encounters
Source: JMIR Med Inform. 2026 Jul 28;14:e88734. doi: 10.2196/88734 (PMC13412140; doi:10.2196/88734)
Supplement: Multimedia Appendix 1 [file medinform-v14-e88734-s001.docx]

## Multimedia Appendix 1: Supplemental Methods

**Scenario Metrics and Design**

To ensure consistency and clinical realism across the simulated encounters, five scripts were designed to mirror common primary care interactions. Each scenario featured a standardized three-way dialogue between a native Spanish-speaking patient or caregiver, an English-speaking clinician, and a Spanish-English interpreter. The simulated encounters were performed by three male medical students who were native Spanish speakers and of similar age. Each student maintained the same role across all scenarios.

On average, the scripts contained 144 words per encounter, resulting in an average audio playback duration of 1.9 minutes. In pediatric scenarios, the terms “patient” and “caregiver” were used interchangeably to refer to the individual providing the clinical history to the provider.

**Interpreter Error Framework and Context**

The study used 20 deliberate errors, modeled after validated frameworks in the established medical interpretation literature. These errors were categorized into three primary types: omissions (leaving out key clinical facts, such as a symptom), substitutions (replacing a fact with incorrect information, such as changing a medication frequency), and additions (incorporating clinical information not stated by the original speaker).

Figure S1 demonstrates the error context (patient vs clinician) in a graphical manner.


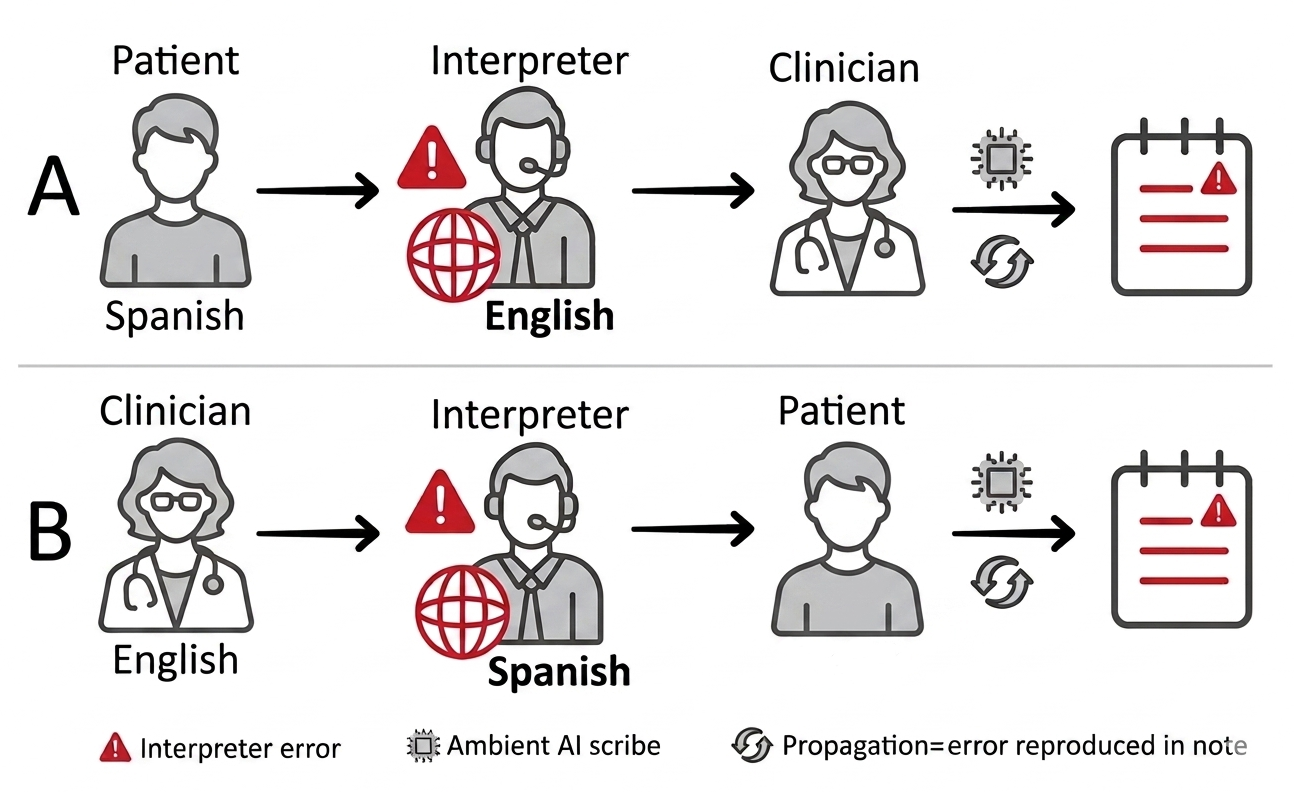


**Figure S1.** Context of interpreter error and propagation. Flow A illustrates an error in which the patient's Spanish speech is misinterpreted into English and documented erroneously by the AI scribe. Flow B illustrates an error in which the clinician's English speech is misinterpreted into Spanish for the patient, with the erroneous version documented in the clinical note. "Patient" also includes the caregiver in pediatric scenarios.
Artificial intelligence-generated image, based on an original author-provided schematic.(Generator: Gemini 3 Flash, Google; May 2, 2026; Requestor: Alexandra Rabotin) [1]

**Technical Specifications and Standardized Environment**

The study was conducted within a standardized, "best-case scenario" acoustic environment to isolate the AI software's processing capabilities from external hardware interference or environmental noise. Audio recordings were played back through the built-in stereo speakers of an HP EliteBook 650 G10 laptop at a fixed volume of 75%. Both ambient AI applications were hosted on an Apple iPhone 14, which was positioned at a standardized distance of 6 inches from the laptop speakers. All audio playback, transcription, and clinical note generation were performed on August 21, 2025.

**Evaluation of Intermediate Outputs**

The intermediate outputs (transcripts) were qualitatively reviewed to understand the models' processing workflow. Both vendors appeared to generate a full bilingual transcript before the final note was generated. Although this review was not systematic, the Spanish and English dialogue appeared to be largely captured during transcription, suggesting that observed discrepancies may have occurred during downstream summarization rather than initial speech recognition.

**References**

1. Google. Gemini 3 Flash generative artificial intelligence model. Generated response to Alexandra Rabotin. May 2, 2026. Accessed May 2, 2026. <https://gemini.google.com/share/90441e707b19>
