# Supplementary material for: Propagation of Interpreter Errors by Ambient AI Scribes: Study Using Simulated Clinical Encounters
Source: JMIR Med Inform. 2026 Jul 28;14:e88734. doi: 10.2196/88734 (PMC13412140; doi:10.2196/88734)
Supplement: Multimedia Appendix 2 [file medinform-v14-e88734-s002.docx]

## Multimedia Appendix 2: Supplemental limitations and suggested considerations for future studies

| **Limitation** | **Suggested consideration for future studies** |
| --- | --- |
| Small sample size and limited number of scripted interpreter errors prevented formal statistical testing; observed differences between vendors, error types, and contexts may be due to chance. Only two vendors assessed. | Use larger scenario sets with enough error instances to support formal statistical comparison across vendors, error types, and encounter contexts. |
| Scenarios were scripted and simulated rather than derived from real-world clinical encounters. | Evaluate AI scribes using real or realistically simulated interpreter-mediated encounters, including natural pauses, interruptions, overlapping speech, and clinically variable dialogue. |
| The scenarios were not created or validated by professional linguists. | Involve professional interpreters, medical linguists, or bilingual communication experts in scenario development and validation to ensure linguistic accuracy, pragmatic realism, and appropriate classification of interpreter error types. |
| Recordings were played back to the AI scribes rather than captured during live conversation, which may affect audio quality and generalizability. | Compare live encounters, audio playback, telemedicine encounters, and different microphone/speaker configurations to determine how capture method affects summarization. |
| The acoustic environment was controlled and likely represented a best-case setting without background noise. | Test performance under real clinical acoustic conditions, including background noise, room echo, masked speech, and overlapping speakers. |
| The study included only two commercially available vendors, and the products were anonymized. | Evaluate additional vendors and, when feasible, report product names, model versions, language settings, and update dates to improve reproducibility. |
| Proprietary AI systems may change over time due to unannounced software or model updates. | Conduct longitudinal audits using the same scenarios at repeated time points to assess model drift and reproducibility. |
| The study focused on final clinical notes and did not systematically analyze intermediate transcripts. | Collect and analyze intermediate transcripts, when available, to distinguish automatic speech recognition failures from downstream natural language processing or summarization failures. |
| Although qualitative review suggested that Spanish and English dialogue were captured in transcription, this was not systematically coded. | Formally compare original scripts, intermediate transcripts, and final notes to identify where errors are introduced, corrected, or amplified. |
| Only Spanish-English encounters were evaluated. | Include additional language pairs, especially languages with fewer training resources, to assess whether error propagation differs by language. |
| The simulated participants were all native Spanish-speaking male medical students of similar age, creating demographic and acoustic homogeneity. | Include speakers with varied sex, age, accents, dialects, speech rates, and vocal characteristics to evaluate speaker diarization and generalizability. |
| The same individuals maintained the same roles across all scenarios. | Rotate speaker roles across scenarios or include multiple actors per role to evaluate whether speaker-specific features influence diarization or summarization. |
| Some scenarios involved caregiver speech for pediatric patients, but caregiver and patient speech were grouped together for analysis. | Separately evaluate adult patient speech, pediatric caregiver speech, and third-party history to determine whether AI scribes weight these sources differently. |
| The study included only three interpreter error types: omissions, substitutions, and additions. | Expand error type to include other common errors such as false fluency and editorialization. |
| Errors were not classified by clinical severity or by section of the encounter. | Stratify errors by clinical severity and location within the note, such as history of present illness, assessment, plan, medication instructions, after visit summary, etc. |
| The study did not include a separate error-free bilingual control scenario. | Include fully accurate bilingual control encounters to establish baseline documentation accuracy before assessing whether models preferentially weight English-language speech. |
| The study did not evaluate whether AI scribes could detect discrepancies between original speech and interpreted speech. | Test whether AI scribes can flag discordance between patient speech and interpreter output and notify clinicians when interpretation discrepancies may affect documentation. |
